# Fungal manipulation of hormone-regulated plant defense

**DOI:** 10.1371/journal.ppat.1006334

**Published:** 2017-06-15

**Authors:** Rajesh N. Patkar, Naweed I. Naqvi

**Affiliations:** 1 Temasek Life Sciences Laboratory and Department of Biological Sciences, National University of Singapore, Singapore; 2 Bharat Chattoo Genome Research Centre, Department of Microbiology and Biotechnology Centre, The Maharaja Sayajirao University of Baroda, Vadodara, India; 3 School of Biological Sciences, Nanyang Technological University, Singapore; The Sainsbury Laboratory, UNITED KINGDOM

## Introduction

Fungi have adapted to diverse habitats and ecological niches, including the complex plant systems. Success of the pathogenic or symbiotic fungi in colonizing the plant tissue depends on their ability to modulate the host defense signaling [1]. Strategies that impart such abilities in fungi include the use of effector proteins that directly disrupt phytohormone-based defense signaling pathways and/or the deployment of mimics of specific plant molecules to evade recognition and the subsequent host immune response [1, 2]. Recent exciting findings have provided insight into a novel strategy whereby the fungal pathogens utilize the endogenous phytohormone-mimics and/or relevant metabolic enzymes to suppress host immunity. These studies strongly suggest that fungal metabolites, in addition to effector proteins, can chemically shape and maintain distinct pathogenic or symbiotic interkingdom relationships between plants and fungi.

## Host-derived hormones as targets of fungal effectors

Fungal pathogens either establish a biotrophic relationship, in which the host plant is kept alive, or are necrotrophic and rampantly kill the invaded host cells to feed on the dead material. Hemibiotrophs begin their invasive lifestyle as biotrophs and, once established, switch to necrotrophy. During host invasion and colonization, fungal pathogens typically target phytohormones such as salicylic acid (SA), jasmonic acid (JA), or ethylene (ET), which are primarily involved in the host defense response, or modulate growth hormones like indole-3-acetic acid (IAA), abscisic acid (ABA), cytokinin (CK), or gibberellin (GA), which can also regulate immune signaling in plants [1]. For instance, the biotrophic fungal pathogen *Ustilago maydis* secretes the chorismate mutase (Cmu1) into the invaded host cells to interfere with the plant SA pathway during disease development in maize [3]. Chorismate is a common substrate/precursor that can be metabolized to amino acids such as phenylalanine or tyrosine via prephenate or utilized in the biosynthesis of SA via isochorismate [4]. Thus, the fungal Cmu1 likely indirectly blocks the synthesis of host SA by channeling the plant-derived chorismate to preferentially produce prephenate (and subsequently the amino acids) instead of isochorismate. Furthermore, *U*. *maydis* also produces a salicylate hydroxylase, Shy1, which degrades the host-derived SA and helps in biotrophic invasion [5]. Interestingly, *SHY1* expression is activated in the presence of SA. Thus, it appears that *U*. *maydis* has a robust strategy to suppress SA-mediated plant immunity by blocking the synthesis of fresh SA while degrading the existing hormonal pool in the host. While *SHY1* orthologs have been reported only in a few fungal genera, chorismate mutase is found in various plant-associated microbes, and its secretion is likely to be a common strategy for modulating the host defense response.

Another biotrophic fungus, *Puccinia graminis* f. sp. *tritici*, expresses a tryptophan 2-monooxygenase (Pgt-IaaM) specifically in the specialized invaginating structure called the haustorium, leading to excess accumulation of host-derived IAA during establishment of the pathogenic interaction in wheat [6].

Several necrotrophic fungi utilize various low-molecular-weight phytotoxic metabolites to influence the accumulation of defense-related plant hormones or employ their own phytohormone-mimics to suppress the plant immunity and/or aid in disease progression [7]. The necrotrophic phytopathogen *Botrytis cinerea* induces the accumulation of a conjugated form of plant IAA (IAA-Aspartate or IAA-Asp) to promote disease development in *Arabidopsis thaliana*. Preliminary studies indicate that the host-derived IAA-Asp supports *in planta* invasive growth by regulating the transcription of virulence genes in the fungal pathogen [8]. IAA-Asp is believed to represent a biologically inactive derivative. Thus, it has been hypothesized that accumulation of host IAA-Asp promotes disease by inducing the expression of specific virulence genes in the phytopathogen rather than via cross talk with other phytohormones or as a direct effect on the overall growth of the fungal pathogen. Interestingly, *B*. *cinerea* also secretes an exopolysaccharide as an elicitor of the host SA pathway to eventually suppress the JA-mediated signaling during invasion of tomato plants [9].

Intriguingly, *Sclerotinia sclerotiorum* has the ability to degrade host-derived SA during necrotrophic growth [10]. However, it remains to be established if such degradation contributes to fungal pathogenesis via modulation of plant immunity during invasive growth. The oomycete *Hyaloperonospora arabidopsidis* attenuates the phytohormone signaling pathways via the RxL44 effector protein, containing the highly conserved arginine (R), a random amino acid (x), leucine (L), and another arginine (R) sequence motif, that targets the host mediator complex subunit MED19a, which is a positive regulator of SA-triggered immunity in *A*. *thaliana* [11]. These findings clearly highlight the distinct strategies evolved by fungal and oomycete pathogens to target/utilize phytohormones to chemically disable plant immunity.

## Fungal phytohormone-mimics as suppressors of plant immunity

**Fig 1:**
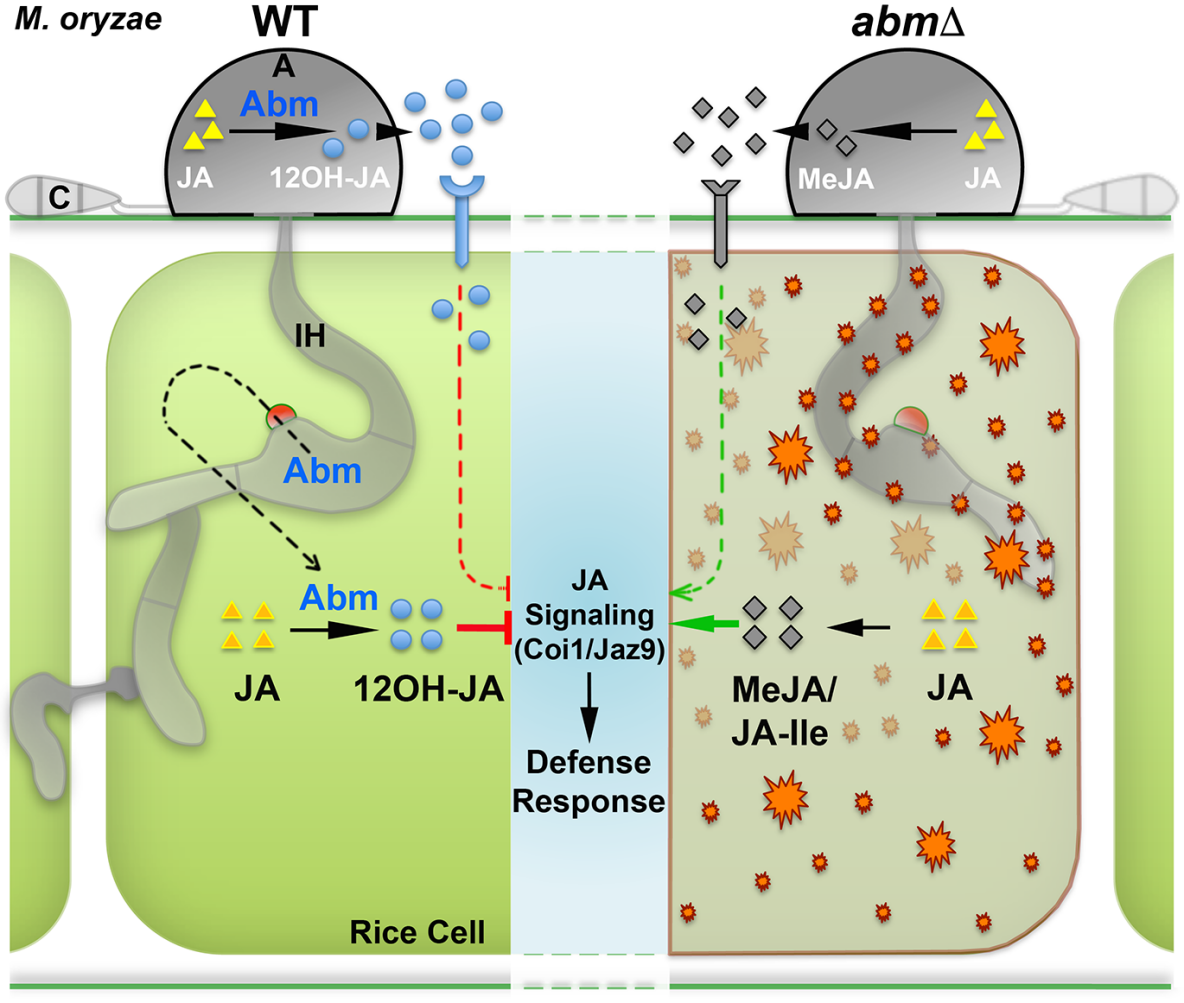
The fungal pathogen *Magnaporthe oryzae* chemically disables the jasmonic acid (JA)–mediated defense signaling in rice. *M*. *oryzae* secretes the antibiotic biosynthesis monooxygenase (Abm) and its endogenously produced chemical effector 12-hydroxy jasmonic acid (12OH-JA) in a biphasic manner to suppress host immunity during establishment of the blast disease in rice. Loss of Abm function leads to activation of the host defense via jasmonate signaling and consequently blocks fungal invasion in rice plants. Brownish orange inclusions depict the sites of methyl JA-induced innate immunity that blocks the *abm*Δ strain of *M*. *oryzae* in the first invaded rice cell. A, appressorium; C, conidium; IH, invasive hypha; JA-Ile, isoleucine conjugate of JA; Jaz9, jasmonate-ZIM domain repressor protein 9; MeJA, methyl JA; WT, wild-type *M*. *oryzae*. Hypothetical receptors or transporters for the fungal JA derivatives have been depicted on the host cell surface. The schematic has not been drawn to scale.

A breakthrough discovery showed how the rice-blast fungus *Magnaporthe oryzae* produces and secretes an analog of a phytohormone to modulate host immunity [12]. The antibiotic biosynthesis monooxygenase (Abm) in *M*. *oryzae* converts intrinsically produced as well as host-derived JA into 12-hydroxyjasmonic acid (12OH-JA) during establishment of the blast disease in rice ([12]; Fig 1). Secreted fungal 12OH-JA blocks JA-mediated signaling (which typically involves perception of jasmonate by the plant F-box protein Coi1 and subsequent degradation of the target repressor Jaz9 [13]) to suppress the defense response during host penetration and the biotrophic growth thereafter. In the absence of the Abm function, *M*. *oryzae* accumulates methyl JA (MeJA), which strongly induces the defense response in rice plants (Fig 1). Interestingly, fungal 12OH-JA and Abm are secreted before and after host penetration, respectively. This suggests that the fungal hydroxylated JA acts as an effector metabolite and helps *M*. *oryzae* in preparing the host for successful entry, while the monooxygenase serves as an effector peptide that aids subsequent tissue colonization (Fig 1). Plants are known to produce 12OH-JA [14]; however, the corresponding enzyme involved in the synthesis of hydroxylated JA in plants has remained elusive thus far. Interestingly, orthologs of *M*. *oryzae ABM* have been found only in several symbiotic bacterial species, suggesting that the blast pathogen lineage acquired *ABM* likely via horizontal gene transfer from rhizosphere bacteria. Phytopathogens such as *Fusarium oxysporum* and *Aspergillus flavus* are known to produce oxilipins, including the plant JA mimics [15]. However, the absence of clear orthologs of *M*. *oryzae ABM* in these fungal pathogens makes it intriguing whether such fungal oxylipins are tailored differently, if at all, to evade or suppress the host immune response.

In addition to JA, *M*. *oryzae* also produces CK using the Cytokinin Synthesis 1 (*CKS1*) [16]. The inability to produce CK, specifically, impairs the *in planta* growth in the *CKS1* mutant due to a precocious induction of the defense response in the invaded rice leaves. It was found that the fungus-derived CKs were likely involved in dampening the host immunity and in mobilizing host nutrients at the site of invasion [16]. *M*. *oryzae* has the ability to produce ABA, too. A recent study showed that the endogenously produced ABA helps *M*. *oryzae* in proper pathogenic development and host invasion, likely via the suppression of plant immunity [17].

These studies highlight the chemical arms race that has shaped the interaction between *M*. *oryzae* and rice [12, 16] in conjunction with stage-specific fungal development and metabolism [18–21].

Just like the pathogenic counterparts, the symbiotic fungi, too, secrete effectors and/or host-mimic molecules to modulate the plant defense machinery to establish mutually beneficial associations [22]. The secreted effector MiSSP7 (Mycorrhiza-induced Small Secreted Protein 7) from *Laccaria bicolor* interacts with PtJaz6, which is a negative regulator of JA-induced gene expression in poplar trees [23]. Thus, MiSSP7 blocks JA-mediated defense signaling by preventing degradation of the PtJaz6 repressor in the host. It would be interesting to assess whether Abm could functionally replace MiSSP7 in *L*. *bicolor* and if 12OH-JA, the aforementioned metabolite from *M*. *oryzae*, targets a Jaz-like repressor of JA signaling in rice.

Similarly, 2 separate studies suggest that down-regulation of the ET pathway by the arbuscular mycorrhizal (AM) fungus is crucial for the establishment of symbiotic associations in the roots [24–26]. Indeed, the AM fungus *Glomus intraradices* suppresses ET signaling by secreting SP7, which directly targets the ET-responsive transcription factor ERF19 [27]. Intriguingly, the ectomycorrhizal truffle species *Tuber melanosporum* and *T*. *borchii* produce endogenous ET, which likely manipulates the hormonal signaling in the host and thus induces morphological changes in the roots [28]. It is possible that such ectomycorrhiza-derived ET could also be involved in modulating the host immunity during establishment of the symbiotic interaction.

The emerging areas of metabogenomics would further help in revealing the secrets of the chemical communication between plant hosts and fungi. Overall, there is a great potential for new and/or as-yet-uncharacterized chemical molecules at the fungus-host interface, for improving the growth potential and inducing disease resistance in plants, and for agribiotech applications in precision agriculture.

## References

[1] Kazan K, Lyons R. Intervention of Phytohormone Pathways by Pathogen Effectors. Plant Cell. 2014;26(6):2285–309. 10.1105/tpc.114.125419 ; 24920334 PMC4114936

[2] Chanclud E, Morel JB. Plant hormones: a fungal point of view. Mol Plant Pathol. 2016;17(8):1289–97. 10.1111/mpp.12393 . 26950404 PMC6638337

[3] Djamei A, Schipper K, Rabe F, Ghosh A, Vincon V, Kahnt J, et al Metabolic priming by a secreted fungal effector. Nature. 2011;478(7369):395–8. 10.1038/nature10454 . 21976020

[4] Chen Z, Zheng Z, Huang J, Lai Z, Fan B. Biosynthesis of salicylic acid in plants. Plant Signal Behav. 2009;4(6):493–6. ; 10.4161/psb.4.6.8392 19816125 PMC2688294

[5] Rabe F, Ajami-Rashidi Z, Doehlemann G, Kahmann R, Djamei A. Degradation of the plant defence hormone salicylic acid by the biotrophic fungus Ustilago maydis. Mol Microbiol. 2013;89(1):179–88. 10.1111/mmi.12269 . 23692401

[6] Yin C, Park JJ, Gang DR, Hulbert SH. Characterization of a tryptophan 2-monooxygenase gene from Puccinia graminis f. sp. tritici involved in auxin biosynthesis and rust pathogenicity. Mol Plant Microbe Interact. 2014;27(3):227–35. 10.1094/MPMI-09-13-0289-FI . 24350783

[7] Prins TW, Tudzynski P, Von Tiedemann A, Tudzynski B, Ten Have A, Hansen ME, et al Infection strategies of Botrytis cinerea and related necrotrophic pathogens In: Kronstad J, editor. Fungal Pathology: Kluwer Academic Publishers; 2000 p. 33–64.

[8] Gonzalez-Lamothe R, El Oirdi M, Brisson N, Bouarab K. The conjugated auxin indole-3-acetic acid-aspartic acid promotes plant disease development. Plant Cell. 2012;24(2):762–77. 10.1105/tpc.111.095190 ; 22374398 PMC3315245

[9] El Oirdi M, El Rahman TA, Rigano L, El Hadrami A, Rodriguez MC, Daayf F, et al Botrytis cinerea manipulates the antagonistic effects between immune pathways to promote disease development in tomato. Plant Cell. 2011;23(6):2405–21. 10.1105/tpc.111.083394 ; 21665999 PMC3160041

[10] Penn CD, Daniel SL. Salicylate degradation by the fungal plant pathogen Sclerotinia sclerotiorum. Curr Microbiol. 2013;67(2):218–25. 10.1007/s00284-013-0349-y . 23512122

[11] Caillaud MC, Asai S, Rallapalli G, Piquerez S, Fabro G, Jones JD. A downy mildew effector attenuates salicylic acid-triggered immunity in Arabidopsis by interacting with the host mediator complex. PLoS Biol. 2013;11(12):e1001732 10.1371/journal.pbio.1001732 ; 24339748 PMC3858237

[12] Patkar RN, Benke PI, Qu Z, Chen YY, Yang F, Swarup S, et al A fungal monooxygenase-derived jasmonate attenuates host innate immunity. Nat Chem Biol. 2015;11(9):733–40. 10.1038/nchembio.1885 . 26258762

[13] Pauwels L, Goossens A. The JAZ proteins: a crucial interface in the jasmonate signaling cascade. Plant Cell. 2011;23(9):3089–100. 10.1105/tpc.111.089300 ; 21963667 PMC3203442

[14] Miersch O, Neumerkel J, Dippe M, Stenzel I, Wasternack C. Hydroxylated jasmonates are commonly occurring metabolites of jasmonic acid and contribute to a partial switch-off in jasmonate signaling. New Phytol. 2008;177(1):114–27. 10.1111/j.1469-8137.2007.02252.x . 17995915

[15] Fischer GJ, Keller NP. Production of cross-kingdom oxylipins by pathogenic fungi: An update on their role in development and pathogenicity. J Microbiol. 2016;54(3):254–64. 10.1007/s12275-016-5620-z ; 26920885 PMC5107414

[16] Chanclud E, Kisiala A, Emery NR, Chalvon V, Ducasse A, Romiti-Michel C, et al Cytokinin Production by the Rice Blast Fungus Is a Pivotal Requirement for Full Virulence. PLoS Pathog. 2016;12(2):e1005457 10.1371/journal.ppat.1005457 ; 26900703 PMC4765853

[17] Spence CA, Lakshmanan V, Donofrio N, Bais HP. Crucial Roles of Abscisic Acid Biogenesis in Virulence of Rice Blast Fungus Magnaporthe oryzae. Front Plant Sci. 2015;6:1082 10.3389/fpls.2015.01082 ; 26648962 PMC4664623

[18] Deng YZ, Qu Z, He Y, Naqvi NI. Sorting nexin Snx41 is essential for conidiation and mediates glutathione-based antioxidant defense during invasive growth in Magnaporthe oryzae. Autophagy. 2012;8(7):1058–70. 10.4161/auto.20217 ; 22561104 PMC3429543

[19] Patkar RN, Ramos-Pamplona M, Gupta AP, Fan Y, Naqvi NI. Mitochondrial beta-oxidation regulates organellar integrity and is necessary for conidial germination and invasive growth in Magnaporthe oryzae. Mol Microbiol. 2012;86(6):1345–63. 10.1111/mmi.12060 . 23043393

[20] Patkar RN, Xue YK, Shui G, Wenk MR, Naqvi NI. Abc3-mediated efflux of an endogenous digoxin-like steroidal glycoside by Magnaporthe oryzae is necessary for host invasion during blast disease. PLoS Pathog. 2012;8(8):e1002888 10.1371/journal.ppat.1002888 ; 22927822 PMC3426555

[21] Ramos-Pamplona M, Naqvi NI. Host invasion during rice-blast disease requires carnitine-dependent transport of peroxisomal acetyl-CoA. Mol Microbiol. 2006;61(1):61–75. 10.1111/j.1365-2958.2006.05194.x . 16824095

[22] Sanders IR. Mycorrhizal symbioses: how to be seen as a good fungus. Curr Biol. 2011;21(14):R550–2. 10.1016/j.cub.2011.06.022 . 21783035

[23] Plett JM, Daguerre Y, Wittulsky S, Vayssieres A, Deveau A, Melton SJ, et al Effector MiSSP7 of the mutualistic fungus Laccaria bicolor stabilizes the Populus JAZ6 protein and represses jasmonic acid (JA) responsive genes. Proc Natl Acad Sci U S A. 2014;111(22):8299–304. 10.1073/pnas.1322671111 ; 24847068 PMC4050555

[24] Geil RD, Peterson LR, Guinel FC. Morphological alterations of pea (Pisum sativum cv. Sparkle) arbuscular mycorrhizas as a result of exogenous ethylene treatment. Mycorrhiza. 2001;11(3):137–43. 10.1007/s005720100120 . 24595433

[25] McArthur DA, Knowles NR. Resistance Responses of Potato to Vesicular-Arbuscular Mycorrhizal Fungi under Varying Abiotic Phosphorus Levels. Plant Physiol. 1992;100(1):341–51. ; 16652967 10.1104/pp.100.1.341 PMC1075557

[26] Zsögön A, Lambais MR, Benedito VA, Figueira AV, Peres LEP. Reduced arbuscular mycorrhizal colonization in tomato ethylene mutants. Scientia Agricola. 2008;65(3):259–67.

[27] Kloppholz S, Kuhn H, Requena N. A secreted fungal effector of Glomus intraradices promotes symbiotic biotrophy. Curr Biol. 2011;21(14):1204–9. 10.1016/j.cub.2011.06.044 . 21757354

[28] Splivallo R, Fischer U, Gobel C, Feussner I, Karlovsky P. Truffles regulate plant root morphogenesis via the production of auxin and ethylene. Plant Physiol. 2009;150(4):2018–29. 10.1104/pp.109.141325 ; 19535471 PMC2719122

